# Epigenetic age is a cell‐intrinsic property in transplanted human hematopoietic cells

**DOI:** 10.1111/acel.12897

**Published:** 2019-02-02

**Authors:** Arne Søraas, Mieko Matsuyama, Marcos de Lima, David Wald, Jochen Buechner, Tobias Gedde‐Dahl, Camilla Lund Søraas, Brian Chen, Luigi Ferrucci, John Arne Dahl, Steve Horvath, Shigemi Matsuyama

**Affiliations:** ^1^ Department of Microbiology Oslo University Hospital Oslo Norway; ^2^ Division of Hematology/Oncology, Department of Medicine, School of Medicine Case Western Reserve University Cleveland Ohio; ^3^ Stem Cell Transplant Program, University Hospitals of Cleveland Case Western Reserve University Cleveland Ohio; ^4^ Department of Pathology Case Western Reserve University Cleveland Ohio; ^5^ Department of Pediatric Hematology and Oncology Oslo University Hospital Oslo Norway; ^6^ Department of Hematology Oslo University Hospital Oslo Norway; ^7^ Institute of Clinical Medicine University of Oslo Oslo Norway; ^8^ Department of Cardiology Oslo University Hospital Oslo Norway; ^9^ National Institute of Aging (NIA) National Institute of Health Bethesda Maryland; ^10^ Department of Human Genetics, David Geffen School of Medicine University of California, Los Angeles Los Angeles California; ^11^ Department of Biostatistics, Fielding School of Public Health University of California, Los Angeles Los Angeles California; ^12^ Case Comprehensive Cancer Center Case Western Reserve University Cleveland Ohio

**Keywords:** aging, DNA methylation, epigenetic clock, glanulocyte-colony stimulating- factor (G-CSF), hematopietic stem cell transfer (HSCT)

## Abstract

The age of tissues and cells can be accurately estimated by DNA methylation analysis. The multitissue DNA methylation (DNAm) age predictor combines the DNAm levels of 353 CpG dinucleotides to arrive at an age estimate referred to as DNAm age. Recent studies based on short‐term observations showed that the DNAm age of reconstituted blood following allogeneic hematopoietic stem cell transplantation (HSCT) reflects the age of the donor. However, it is not known whether the DNAm age of donor blood remains independent of the recipient's age over the long term. Importantly, long‐term studies including child recipients have the potential to clearly reveal whether DNAm age is cell‐intrinsic or whether it is modulated by extracellular cues in vivo. Here, we address this question by analyzing blood methylation data from HSCT donor and recipient pairs who greatly differed in chronological age (age differences between 1 and 49 years). We found that the DNAm age of the reconstituted blood was not influenced by the recipient's age, even 17 years after HSCT, in individuals without relapse of their hematologic disorder. However, the DNAm age of recipients with relapse of leukemia was unstable. These data are consistent with our previous findings concerning the abnormal DNAm age of cancer cells, and it can potentially be exploited to monitor the health of HSCT recipients. Our data demonstrate that transplanted human hematopoietic stem cells have an intrinsic DNAm age that is unaffected by the environment in a recipient of a different age.

## 1 INTRODUCTION

Several publications describe DNA methylation (DNAm)‐based biomarkers of aging which can be used to estimate the age of a tissue (Hannum et al., 2013; Horvath, 2013; Spolnicka et al., 2016; Weidner & Wagner, 2014). For example, the multitissue age estimator utilizes the weighted average of 353 CpG sites to arrive at an age estimate that is referred to as DNAm age (Horvath, 2013). Age‐adjusted measures of DNAm age are predictive of life span (Chen et al., 2016; Marioni et al., 2015) and relate to a host of conditions, including obesity (Horvath et al., 2014), HIV infection (Horvath & Levine, 2015), Down syndrome (Horvath et al., 2015), Parkinson's disease (Horvath & Ritz, 2015), Werner syndrome (Maierhofer et al., 2017), and menopause (Carroll et al., 2017). Lifestyle factors have only a weak effect on the DNAm age of blood (Quach et al., 2017), suggesting that DNAm age largely reflects cell‐intrinsic properties. It is not yet known to what extent secreted factors (e.g., hormones, cytokines, growth factors, and metabolites) from other organs affect the DNAm age of blood, or whether DNAm age is a cell‐intrinsic feature.

To address this question, we analyzed blood samples from allogeneic hematopoietic stem cell transplantation (HSCT) recipients. Allogeneic HSCT is an effective treatment for leukemia (Thomas et al., 1979; Thomas, Lochte, Lu, & Ferrebee, 1957). In HSCT, the patient's original hematopoietic stem cells (HSCs) are eradicated (using ablative chemotherapy and/or radiotherapy) and subsequently replaced by healthy HSCs from a donor (obtained via bone marrow (BM) aspiration or granulocyte‐colony‐stimulating factor (G‐CSF)‐stimulated leukapheresis; Dreger et al., 1993; Russell, Hunter, Rogers, Hanley, & Anderson, 1993). If the treatment is successful, the donor cells will engraft in the recipient's BM and go on to reconstitute the entire hematopoietic system, including white blood cells, red blood cells, and platelets. After transplantation, the donor cells will thus be exposed to the environment of the recipient for many years.

Experiments involving heterochronic parabiosis or the transfer of factors from human cord blood to old mice have demonstrated that factors present in the younger blood might rejuvenate older tissues (Castellano et al., 2017; Conboy, Conboy, & Rando, 2013; Eggel & Wyss‐Coray, 2014). On the other hand, recently published work suggests that the DNAm age of transplanted blood cells is maintained at the donor's age, at least under short‐term observations (Spolnicka et al., 2016; Stölzel et al., 2017; Weidner et al., 2015). However, it is not yet known if the DNAm age of the donor cells is affected by the recipient's age after prolonged exposure to the recipient's signaling environment. The aforementioned DNAm studies have limitations regarding their feasibility for identifying a potential rejuvenating effect on donor cells after long‐term exposure to a younger environment. Therefore, in an ethically acceptable human in vivo setting, the key question regarding age rejuvenation has still not been adequately addressed. The previous studies have one or more of the following unexamined issues. First, most of the donor–recipient pairs were of roughly the same age or the donor was younger than the recipient. Second, HSC donors were mostly younger than the recipients as opposed to the other way around. Third, Spolnicka et al. (2016) and Weidner et al. (2015) used blood cell‐specific DNAm age estimators, but not the multitissue DNAm age estimator. Fourth, children or adolescents were not included in any of these studies. Finally, these studies had a short follow‐up time, with a mean of 126 days, 1 year, and up to 8.8 years (Spolnicka et al., 2016; Stölzel et al., 2017; Weidner et al., 2015). The study by Stolzel et al. involved recipients who were followed up for more than 12 months; however, the age difference between donor and recipient was not reported. This is an important aspect, as factors in the plasma of human cord blood were reported to have a rejuvenating effect in a human–animal study (Castellano et al., 2017).

The present study was designed to test two competing hypotheses: (a) DNAm age of hematopoietic cells is a cell‐intrinsic property that is not influenced by factors in the stem cell niche and non‐hematopoietic tissues in the human body, and (b) DNAm age of hematopoietic cells is determined through interactions with the stem cell niche and other cell types in the human body. To this end, we overcame the key limitations of previous studies by (a) analyzing several donor–recipient pairs with a substantial age difference (1–49 years), (b) including young children, and (c) including long follow‐up times.

Here, we report that, despite a substantial age difference between donor and recipient, the DNAm age of transplanted donor blood reflects the age of the donor, even after many years of exposure to the recipient's body. This observation was consistent for both adult and child recipients. Our data demonstrate that the DNAm age of transplanted blood cells is cell‐intrinsic in the human body.

## 2 RESULTS

### 2.1 Study population

**Figure 1:**
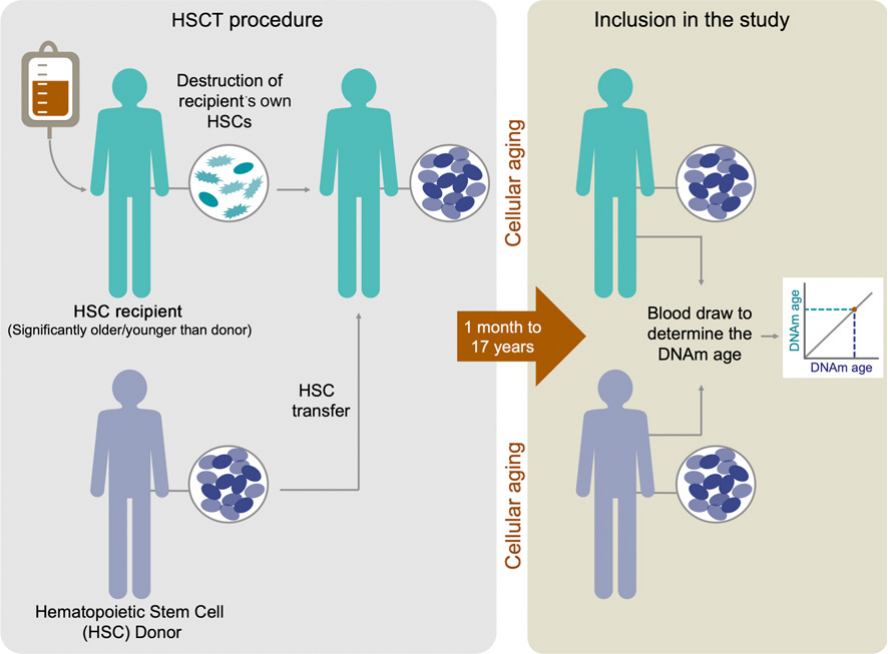
Schematic explanation of the study design. Blood was collected from recipients between 1 month and 17 years after HSC transplantation (HSCT). Donor chimerism and DNAm age were measured. Donor–recipient pairs with a large age difference (1–49 years) were included

**Figure 2:**
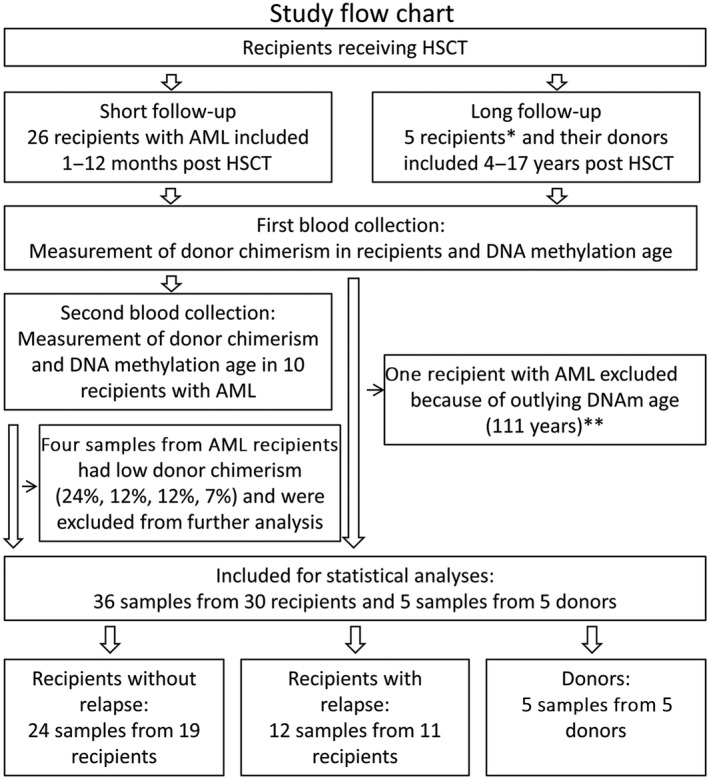
Study flow chart with exclusions. Ten recipients were sampled twice, and ten others were sampled once. Five recipients were excluded because of low donor chimerism. * Three recipients had hematological cancer, while two had other indications. **In addition, one sample that was a statistical outlier (1.5 times the interquartile range above the third quartile) with a DNAm age of 111 years (donor chimerism was 97%) was excluded from the analyses. The rationale for this exclusion is supported by the fact that the recipient died due to relapse of leukemia within 1 month of blood sampling, implying the presence of an unrecognized health problem at the time of blood collection.

In total, 31 HSC recipients aged 18–74 years were included in the study. Their blood was collected between 1 month and 17 years after HSCT (Figures 1 and 2). The recipients were 2–74 years old at the time of transplantation, and the HSC donors (*n* = 31) were 21–58 years old at the time of donation. Acute myeloid leukemia (AML) was the most common indication for HSCT (*n* = 26), but three recipients had other hematological cancers and two had other indications (Table 1).

**Table 1:** List of DNA samples collected from blood cells of recipients who had received HSCT

Sample ID	Recipient age at blood draw^a^	Donor age at blood draw^a^	DNAm age of recipient^a^	Repeated sample from same recipient	Time of sampling (months after HSCT)	Alive at last follow‐up	Relapsed	Age difference recipient‐donor	Difference: Recipient DNAm age—recipient age	Difference: Recipient DNAm age—donor age	Recipient age at HSCT	Donor age at HSCT	Donor chimerism (%)	DNAm age of donor	Stem cell source
1	37	59	57	0	204	Yes	No	−22	20	−2	20	42	>99	55	BM
2	20	52	58	0	204	Yes	No	−32	38	6	3	35	>99	53	BM
3	26	60	53	0	144	Yes	No	−34	27	−7	14	48	>99	58	PB
4	18	42	46	0	192	Yes	No	−24	28	4	1	26	>99	41	BM
5	27	60	60	0	48	Yes	No	−33	33	0	23	56	>99	66	PB
502	64	26	21	0	1	No	No	38	−43	−5	64	26	96	NA	PB
579	69	25	19	0	1	No	No	44	−50	−6	69	25	97	NA	PB
583	68	58	60	0	2	No	Yes	10	−8	2	68	58	99	NA	PB
647	56	45	33	0	3	Yes	No	11	−23	−12	56	45	100	NA	PB
750	72	29	33	0	1	Yes	No	43	−39	4	72	29	96	NA	PB
753	70	37	29	0	1	No	No	33	−41	−8	70	37	97	NA	PB
806^b^	69	25	111	0	4	No	Yes	44	42	86	69	25	97	NA	PB
833	62	46	43	0	3	Yes	No	16	−19	−3	62	46	99	NA	PB
834	67	28	19	0	2	No	No	39	−48	−9	67	28	100	NA	PB
908	66	34	43	0	4	No	Yes	32	−23	9	66	34	98	NA	PB
915	52	26	23	0	1	No	Yes	26	−29	−3	52	26	99	NA	PB
915 b	52	26	67	1	3	No	Yes	26	15	41	52	26	24	NA	PB
926	74	25	55	0	1	No	Yes	49	−19	30	74	25	88	NA	PB
926 b	74	25	249	1	15	No	Yes	49	175	224	73	24	12	NA	PB
950	50	22	13	0	2	No	Yes	28	−37	−9	50	22	95	NA	PB
950 b	50	22	9	1	11	No	Yes	28	−41	−13	49	21	12	NA	PB
952	66	32	36	0	3	Yes	No	34	−30	4	66	32	100	NA	PB
958	73	31	35	0	1	No	No	42	−38	4	73	31	94	NA	PB
1021	54	30	19	0	1	Yes	Yes	24	−35	−11	54	30	98	NA	PB
1021	54	30	7	1	4	Yes	Yes	24	−47	−23	54	30	98	NA	PB
1176	71	49	40	0	2	No	Yes	22	−31	−9	71	49	98	NA	PB
1176^b^	71	49	78	1	6	No	Yes	22	7	29	71	49	7	NA	PB
1259	32	24	20	0	6	No	Yes	8	−12	−4	32	24	100	NA	PB
1293	54	25	22	0	5	No	Yes	29	−32	−3	54	25	96	NA	PB
1296	62	26	36	0	3	No	Yes	36	−26	10	62	26	100	NA	PB
1318	54	26	19	0	4	No	Yes	28	−35	−7	54	26	97	NA	PB
1007/1363	39	35	38	0	1	Yes	No	4	−1	3	39	35	98	NA	PB
1007/1363	39	35	17	1	12	Yes	No	4	−22	−18	38	34	97	NA	PB
1033/1191	73	51	42	0	1	Yes	No	22	−31	−9	73	51	98	NA	PB
1033/1191	73	51	44	1	7	Yes	No	22	−29	−7	72	50	98	NA	PB
1069/1305	36	35	30	0	3	Yes	No	1	−6	−5	36	35	98	NA	PB
1069/1305	36	35	31	1	9	Yes	No	1	−5	−4	35	34	98	NA	PB
754/812	28	30	31	0	1	Yes	No	−2	3	1	28	30	100	NA	BM
754/812	28	30	27	1	3	Yes	No	−2	−1	−3	28	30	100	NA	PB
878/1016	57	25	20	0	1	Yes	No	32	−37	−5	57	25	100	NA	PB
878/1016	57	25	16	1	7	Yes	No	32	−41	−9	56	24	99	NA	PB

Notes

“Relapsed” indicate whether the recipient relapse was detected at the latest time point when the patient contacted the hospital.

BM, bone marrow; NA: Data not available; PB, peripheral blood.

a Ages are at the time blood was drawn for our study, 1 month to 17 years after HSCT.

b Sample excluded from statistical analyses as an outlier or due to low donor chimerism (<80%).

Recipients were included in the study after giving written informed consent, and blood samples were obtained for determination of DNAm age and donor chimerism. Ten recipients contributed two blood samples each, whereas the remaining 21 recipients contributed one blood sample (Table 1; Figure 2). For the first blood sample of the twice‐sampled recipients, donor chimerism was above 94% in all but one of the recipients (88%). For the second blood sample, six recipients had donor chimerism >97%, whereas four recipients showed low chimerism scores (as low as 24%, 12%, 12%, and 7%), indicating repopulation of the recipient's leukemic cells (Table 1). Therefore, the four samples with low chimerism were excluded from further analysis. In addition, a statistically extreme outlier sample (Sample ID 806 in Table 1; Supporting Information Figure S1a,b) with a DNAm age of 111 years and donor chimerism of 97% was identified. This was from a patient with relapse of leukemia, several viral infections, and acute GVHD grade 3 who died shortly after the sample was obtained. We excluded this sample from the presented statistical analysis, but included it where it was relevant as well as in the discussion because we cannot exclude that it may represent a clinically meaningful rare case. After the above‐listed exclusions, a total of 36 blood samples from 30 recipients were subjected to further analysis.

### 2.2 Differences in DNAm age

**Figure 3:**
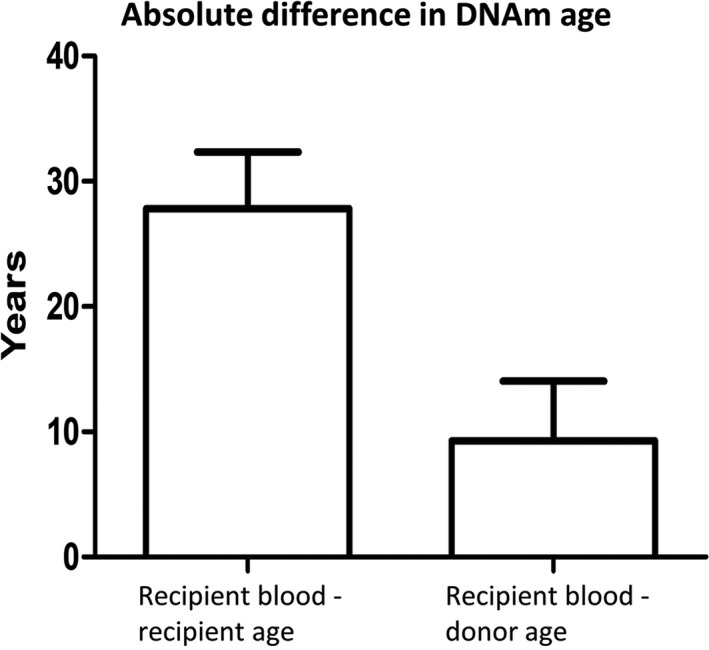
The DNAm age of the recipients’ blood resembles the chronological age of the donors more closely than the recipients. The mean absolute difference between the DNAm age of the recipients’ blood and chronological age of the recipients was 26 years, whereas the difference between the DNAm age of the recipients’ blood and the age of the donor was 7.2 years. This difference between these averages is statistically significant (*t*‐test: *p* < 0.0001, *n* = 36). Error bars show 95% confidence interval of the mean

The mean absolute difference between the DNAm age of the recipients’ blood (1 month to 17 years after transplantation) and the chronological age of the recipient was 27 years (Figure 3). In comparison, the mean absolute difference between the DNAm age of the recipients’ blood and the chronological age of the donor was 7.2 years, which is significantly less than the preceding value (paired *t* test and Wilcoxon test, *p* < 0.0001; Figure 3). This initial comparison suggests that the DNAm age of the recipients’ blood more closely reflects the chronological age of the donor than the chronological age of the recipient.

**Figure 4:**
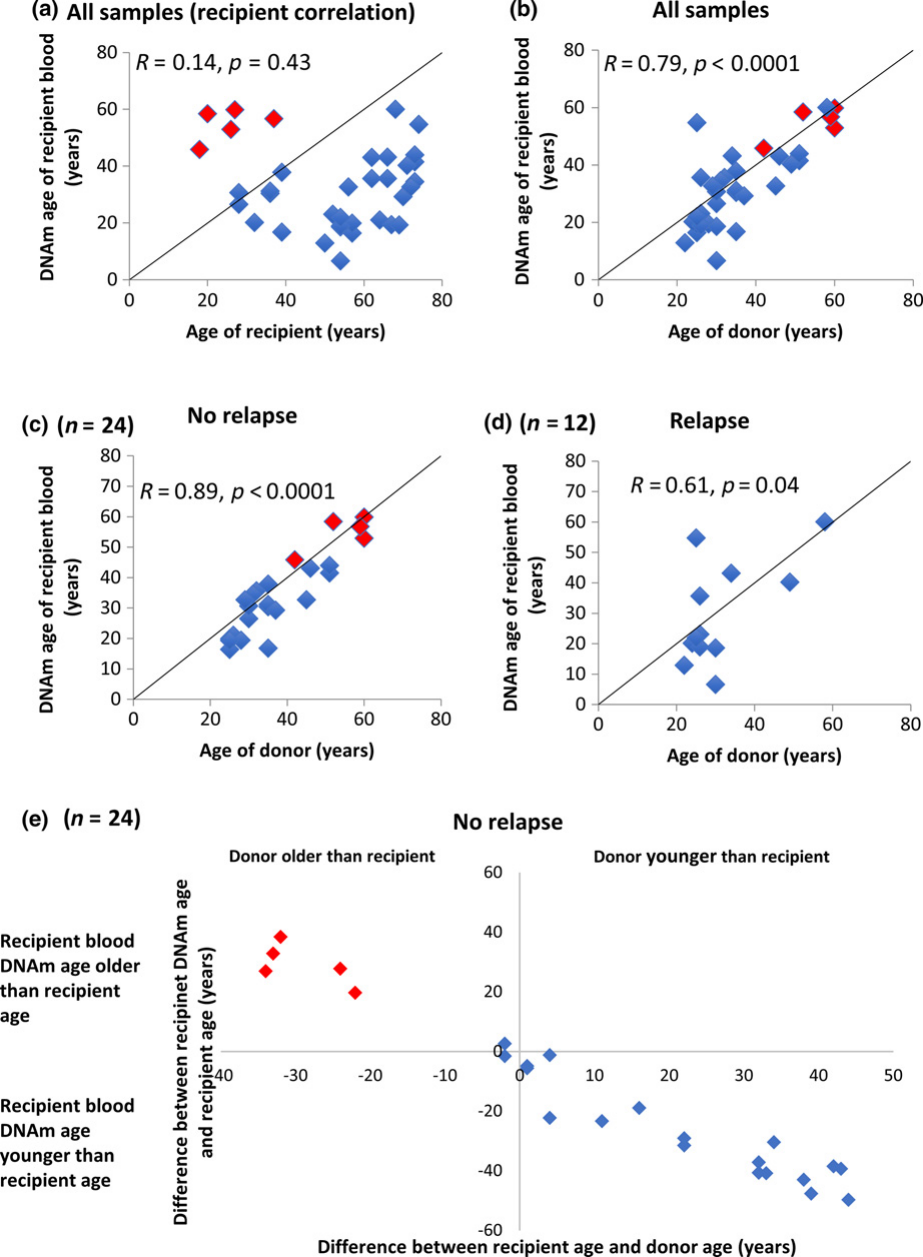
The DNAm age of the recipients’ blood cells correlates with the chronological age of the donor, but not the chronological age of the recipient. In panels (a–f), red dots indicate samples obtained 4–17 years after HSCT, and blue dots indicate samples obtained <1 year after HSCT. (a) The DNAm age of recipients’ blood did not correlate with the chronological age of the recipients when all 36 samples were included (*R* = −0.14, *p* = 0.43, *n* = 36). (b) The DNAm age of the recipients’ blood correlated with the donors’ chronological age when assessing all samples (*R* = 0.79, *p* < 0.0001, *n* = 36). (c) The DNAm age of blood from the recipients without relapse correlated strongly with the chronological age of donors (*R* = 0.89, *p* < 0.0001, *n* = 24). (d) The DNAm age of blood from recipients who had relapse of acute myelogenous leukemia correlated less with the donors’ chronological age (*R* = 0.61, *p* = 0.04, *n* = 12). When the outlier is included, the corresponding values become *R* = 0.22 and *p* = 0.47 (*n* = 13). (e) In the 24 samples from non‐relapsing recipients, the age difference between donor and recipient correlated strongly with the difference between the DNAm age of the recipients’ blood and the recipients’ chronological age (*R* = 0.98, *p* < 0.0001, *n* = 24). The correlation was indifferent to the size or direction of the age difference between recipient and donor. For example, DNAm age was much higher than the recipients’ ages if they received HSCs from donors much older than themselves (upper left quadrant)

To assess how DNAm age relates to the chronological age of recipient and donor, we carried out a correlation analysis. Pearson correlations were calculated and showed that the DNAm age of the recipients’ blood post‐transplantation did not correlate with the chronological age of the recipients (*R* = −0.14, *p* = 0.43, 36 samples from 30 recipients; Figure 4a). Instead, the DNAm age of the recipients’ blood closely correlated with the donors’ chronological age (*R* = 0.79 and *p* < 0.0001; Figure 4b). This correlation was even more pronounced in samples obtained from the 19 recipients who did not experience relapse of AML within the study period (*R* = 0.89, *p* < 0.0001, 24 samples from 19 recipients) and less pronounced in recipients who experienced relapse (*R* = 0.61 and *p* = 0.04, 12 samples from 11 recipients), even though donor chimerism in the latter group was >88% (Figure 4c,d). The outlier (Sample ID 806) did experience relapse of AML, and when included in this calculation, the correlation among relapses was no longer significant (*R* = 0.22 and *p* = 0.47).

The correlations were not influenced by the time since transplantation (Figure 4c) or by the size or direction of the age difference between recipient and donor (Figure 4e).

**Figure 5:**
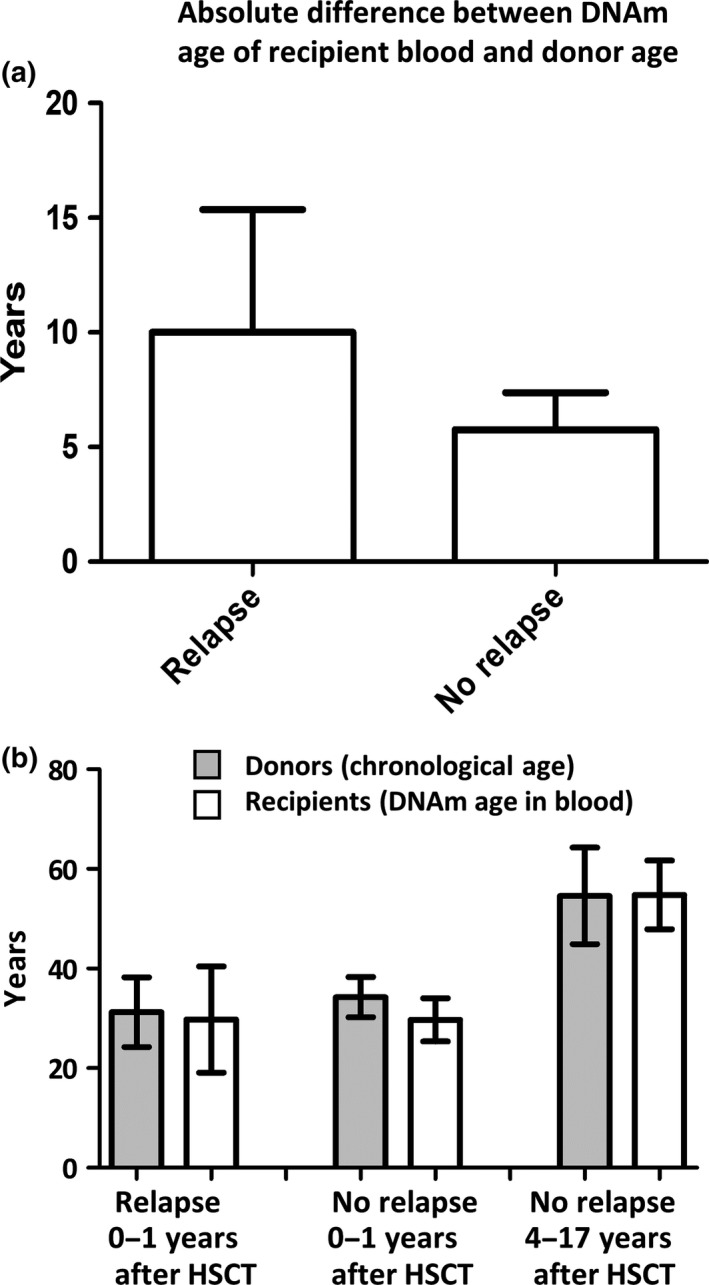
Comparison of the differences between the DNAm age of the recipient blood and donor's age among groups with different patient outcomes. (a) The bar graphs show the absolute difference between the donor's chronological age and the DNAm age of the recipient's blood in the “relapse” and “no relapse” groups. The difference is smaller in the “no relapse” patient group. Detailed patient information is listed in Table 1. (b) DNAm age rejuvenation was observed within 1 year of HSCT. The average donor age and the recipients’ blood DNAm age are shown in three different groups: relapse, no relapse (0–1 years), and no relapse (4–17 years). There was a statistically significant rejuvenation of DNAm age (4.7 years *p* < 0.003) in the “no relapse” (0–1 year) group in comparison with the donors’ chronological age. Detailed patient information is listed in Table 1

The mean absolute difference between the DNAm age of the relapse samples (*n* = 12) and the donor age was 10 years, which was not significantly higher than that of the non‐relapse samples (*n* = 24) at 5.8 years (*t* test, *p* = 0.04 and Mann–Whitney U (MW‐U) test, *p* = 0.12; Figure 5a).

**Figure 6:**
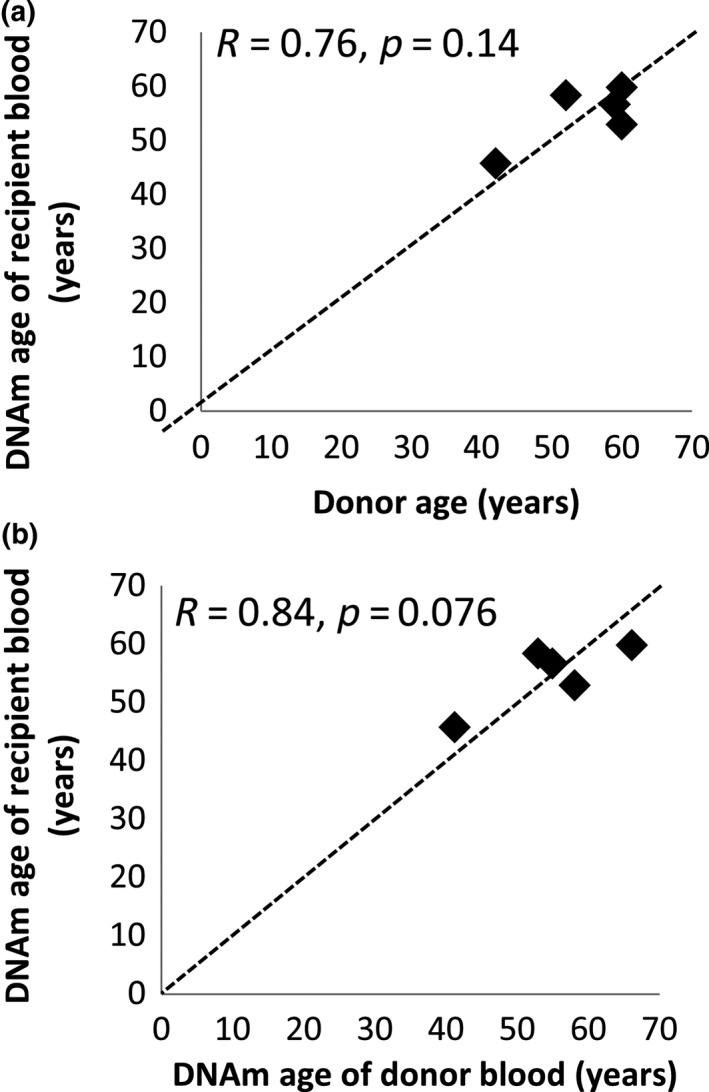
The DNAm age of the recipients’ blood cells correlates with the DNAm age of the donors’ blood cells in the five donor–recipient pairs with donors' blood available. In five HSCT donor–recipient pairs, blood from both the donor and the recipient was available. Blood from these pairs was obtained between 4 and 17 years after HSCT to treat childhood leukemia (*n* = 3) or other hematological disorders (*n* = 2). In these pairs, the DNAm age of the recipients’ blood correlated with the chronological age of the donors (a) (*R* = 0.76, *p* = 0.14, *n* = 5) as well as with the DNAm age of the donors’ blood (b) (*R* = 0.84, *p* = 0.076, *n* = 5) obtained at the same time

When we restricted the analysis to the 24 recipients who did not relapse, we found that samples obtained within a year of HSCT exhibited a statistically significant rejuvenation of DNAm age (4.7 years, *t* test: *p* = 0.003, Wilcoxon test: *p* < 0.004; Figure 5b). In the five participants with >1 year (4–17 years) of follow‐up, donors were also included in the study and contributed blood samples. The DNAm age of the donors’ blood was strongly correlated to both donor age (*R* = 0.84, *p* = 0.08) and the DNAm age of the recipients’ blood (*R* = 0.76, *p* = 0.14; Figure 6a,b).

**Figure 7:**
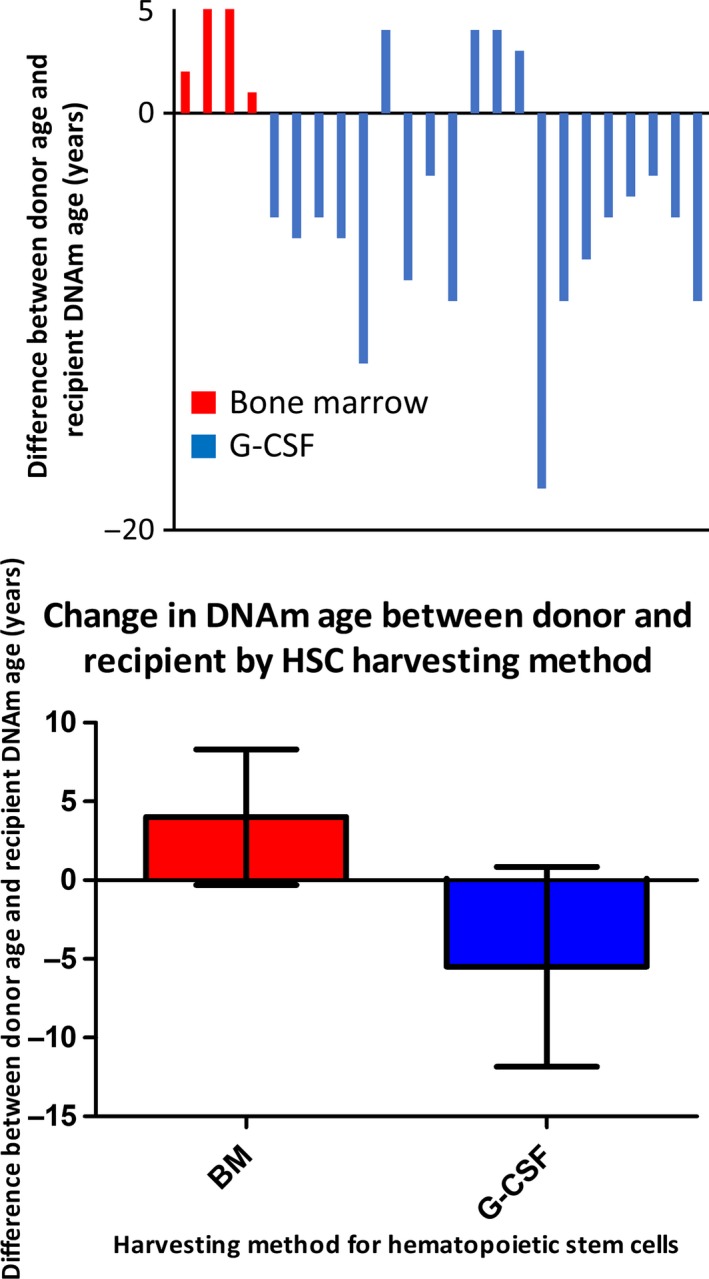
The DNAm age progression of transplanted HSCs may be affected by the HSC harvesting method. (a) This figure depicts the difference between donor age and recipient DNAm age. (The donor's age was given as DNAm age for the five cases shown in Figure 6, and as chronological age for the 19 cases in Table 1, “no relapse” patients. Total *n* = 24.) The red bars indicate the results from patients who received HSCs obtained from donor BM without G‐CSF treatment. The blue bars indicate the results from patients who received HSCs isolated from donor peripheral blood after treatment with G‐CSF. (b) The average difference between donors' age and recipient's DNAm age, from the results shown in panel (a). +3.3 years for the BM method and −5.0 years for the G‐CSF method. Error bars show 95% confidence interval of the mean. The difference is statistically significant, (*p* = 0.01 (*t* test) or *p* = 0.01 (nonparametric MW‐U test))

In theory, the HSC harvesting method might have influenced the DNAm age in the 24 recipients without relapse. In 20 cases, HSCs were harvested from peripheral blood (PB in Table 1) of G‐CSF‐treated donors. In the other four cases, HSCs were obtained directly from bone marrow (“BM” in Table 1). The type of age gap (i.e., positive or negative) between the donors’ age (DNAm age or chronological age) and the recipients’ DNAm age was different between the two methods (Figure 7a). On average, the difference was −5.0 years (standard error (*SE*) = 1.3) for the G‐CSF method and +3.3 years (*SE* = 1.0) for the BM method (Figure 7b). This difference is statistically significant; however, this was an unplanned analysis from which we cannot draw strong conclusions (*t* test, *p* = 0.01; and MW‐U test, *p* = 0.01).

## 3 DISCUSSION

The present study shows that the DNAm age of donor blood is not influenced by the environment of the recipient's body, whether younger or older, and that the DNAm age continues to increase after transfer to the recipient's body as if the donor cells were still in the donor's body. This trait persisted even 17 years after the transfer to recipients who were 1 and 3 years old at the time of HSCT. This suggests that the DNAm age of human hematopoietic cells is not affected by BM niche cells or other factors in the recipient's body. We can therefore conclude that epigenetic age is a cell‐intrinsic property in transplanted human hematopoietic cells.

Our observation is consistent with previous studies examining other types of age‐dependent DNAm levels in hematopoietic cells (Spolnicka et al., 2016; Weidner et al., 2015). In these previous studies, three (Weidner et al., 2015) or five (Spolnicka et al., 2016) CpG sites were analyzed after 4 months or 1 year after HSCT. Stölzel et al. also used the same multitissue DNAm age estimator that we used in the present study, but they did not report the age difference between donors and recipients and only analyzed blood samples collected within 8 years after HSCT (Stölzel et al., 2017). In contrast, we analyzed blood samples collected up to 17 years after HSCT from recipients who had much older or younger donors. Through access to the Norwegian national records of child HSCT, we were able to identify five pairs of pediatric patients (children and adolescents) and adult donors who were willing to participate in this study. These patients received HSCT between 4 and 17 years before their blood samples were collected for this study. The contributions from these five pairs allowed us to compare the DNAm age of blood from both donors and recipients. Our study adds to an increasing body of literature demonstrating that the DNAm age of hematopoietic cells progresses independently from other tissues or cell types in humans. Thus, from the viewpoint of DNAm‐based biomarkers of aging, rejuvenation of hematopoietic cells does not occur, even when HSCs from adults grow and differentiate in a child's or adolescent's BM niche for up to 17 years. However, our finding does not exclude the possibility that other age‐related changes of blood cells may be influenced by interaction with the younger environment. A possible caveat to our study is that the patients received myeloablative conditioning regimens. Since these regimens will alter the physiology of the BM niche, we cannot exclude the possibility that these treatments influenced the progression of the DNAm age of the blood cells transplanted into the recipients (Hooper et al., 2009). It is also important to state as a possibility that the transplanted HSCs influenced the DNAm age of the recipient cells. Further studies are needed to examine these important remaining questions.

We monitored time‐dependent changes of the DNAm age of blood after HSCT (Table 1; Figure 5). In some cases, especially in recipients who experienced relapse of leukemia, the DNAm age of the blood was unstable, probably due to the gradual repopulation by leukemia cells. For example, DNAm age was abnormally accelerated or rejuvenated in blood with low chimerism percentage scores; that is, the recipient's cancer cells repopulated in these patients (Table 1; e.g., Sample ID 926, 950, 1021, and 1176). In contrast, the DNAm age of transplanted blood was maintained and aged normally for up to 17 years in the recipients who remained in remission (Table 1; e.g., Sample ID 1–5; Figure 3). These results support those of other studies demonstrating that epigenetic age acceleration in blood is predictive of cancer (Ambatipudi et al., 2017; Dugué et al., 2018; Horvath, 2013; Levine et al., 2015). In our study, there was an outlier with high donor chimerism (97%) that showed an accelerated DNAm age of 111 years in a 69‐year‐old recipient with HSCs obtained from a 25‐year‐old donor. Although this was a rare case (1 outlier in 37 samples), it suggests that the mechanisms affecting DNAm age in the transplantation setting may not be the same in all donor/recipient settings. The outlier might also be an extreme example of the observed drift in DNAm age that was seen to affect other patients experiencing relapse of leukemia. Inclusion of this sample in the analyses did not change any conclusions in this study except for the correlation between donor age and DNAm age of recipients with relapse, which switched from being statistically significant to non‐significant (*R* = 0.22, *p* = 0.47). Our limited data set does not allow us to conclude whether DNAm age can be used as a predictive biomarker for leukemia relapse or other health problems following HSCT; however, our data encourage further work to follow up on this. Future prospective large‐scale studies with detailed outcome data are warranted to determine whether DNAm age analysis is useful for prediction of leukemia relapse after HSCT.

We observed a rejuvenation of DNAm age in the blood of the “no relapse” recipients within 1 year after HSCT (Figure 5), which is consistent with the study by Stölzel et al. (2017). Stölzel et al. also reported that accelerated epigenetic aging was observed more than 6 months after HSCT (2.4 years per chronological year up to 8 years after HSCT; Stölzel et al., 2017). However, we did not observe a significant DNAm age acceleration (or rejuvenation) in our long‐term follow‐up analysis (Figure 5). This difference between the two studies may be due to the different treatment strategies (e.g., selection of chemotherapies) or reflect technical differences (e.g., DNA storage conditions, bisulfite conversion, or different DNAm normalization methods).

Our study advances understanding of the mechanism of the epigenetic clock by having addressed questions left unanswered in previous studies of the DNAm age of blood cells after HSCT. We established, for the first time, the long‐term (up to 17 years) effects of HSCT on DNAm age of blood cells transplanted from healthy donors 1–49 years younger or older than the recipients. The results suggest that the child's (recipient's) body did not rejuvenate the DNAm age of adult blood cells. Notably, we were able to determine the DNAm age of blood cells from both donor and recipient samples collected at the same time for at least five pairs of childhood/adolescent HSCT cases, which strengthens our main conclusion that the epigenetic clock of a donor's blood cells progresses at the same speed in both the donor's body and the recipient's body. Finally, our study included a total of 26 donor–recipient pairs with a 1‐ to 49‐year difference in age, making it the largest study of its kind. In addition, the unplanned analyses shown in Figure 7 suggest that G‐CSF may have an ability to rejuvenate DNAm age of HSCs. G‐CSF has a pronounced influence on cellular processes in HSCs and has been shown to selectively mobilize dormant HSCs to the bloodstream in mice (Bernitz, Daniel, Fstkchyan, & Moore, 2017; Panch, Szymanski, Savani, & Stroncek, 2017). In our study, these processes could potentially have influenced the DNAm age of the transplanted HSCs or their progeny either directly or through selection by mobilization, and thus selected HSCs with a lower DNAm age. In the BM aspiration cases, G‐CSF was not applied and these processes would not have been active, explaining the lack of rejuvenation here. Further investigation with a larger sample size is necessary to draw a robust conclusion about the effects of G‐CSF.

In summary, the present study suggests that the DNAm age of hematopoietic cells progresses independently from other cell types in the human body. If there is an influence on DNAm age between hematopoietic cells and other cells, it will be a unidirectional one from hematopoietic cells to other cell types. However, the present study was performed by analyzing blood cells donated from patients who had received myeloablative conditioning regimens that may have an impact on the epigenetic clock system of the recipient's BM niche. Further studies are necessary to understand the mechanism governing how the DNAm ages of various tissues are coordinated in the human body.

## 4 EXPERIMENTAL PROCEDURES

### 4.1 Blood from HSCT recipients and donors (*n* = 26), follow‐up <1 year

For the analyses of the DNAm age of blood from the 26 adult AML patients, blood samples were obtained from the Hematopoietic Biorepository and Cellular Therapy Facility at Case Western Reserve University. Written informed consent was received from participants prior to inclusion in the study. The University Hospitals Cleveland Medical Center IRB approved the human subject research.

### 4.2 Blood from patients treated for childhood/young adult leukemia or other hematopoietic disorders (*n* = 5), follow‐up 4–17 years

Patient and donor data were retrieved from the database of transplanted patients going back 20 years at the large transplantation center at Oslo University Hospital, Norway. Of 16 donor–recipient pairs fulfilling the criteria that (a) the donor was >10 years older than the recipient, (b) both donor and recipient were still alive and willing to contribute blood to the study, and (c) had >4 years of follow‐up, only five pairs agreed to participate. Patients and their donors were included in the study after written informed consent, and the study was approved by the Regional Committee for Research Ethics (REK 2015/1849). Peripheral blood samples (10 ml) were collected in EDTA tubes, snap frozen on dry ice (−78.5), and stored at −80 until DNA extraction. Samples from patients (recipients) and their donors were collected on the same day.

### 4.3 DNAm analysis and epigenetic clock analysis

All DNAm analyses were performed with the Illumina Infinium 450 K platform in the core facility at UCLA as previously reported (Horvath & Levine, 2015). Genomic DNA extraction and STR PCR were performed as reported (Thiede et al., 1999). CpG methylation analysis was performed using Illumina BeadChip arrays (Illumina, San Diego, USA). DNAm age was estimated using the published algorithm with Noob normalization (Horvath, 2013).

## CONFLICT OF INTEREST

The authors declare no conflicts of interest.

## AUTHORS’ CONTRIBUTIONS

Arne Soraas and Mieko Matsuyama designed the experiments, performed the majority of the experiments. Marcos de Lima, David Wald, Jochen Buchner, and Tobias Gedde‐Dahl collected blood samples and managed recipients’ clinical information. Camilla Søraas contributed to manuscript writing and statistical analysis. Brian Chen and Luigi Ferrucci participated in the study design and data analysis. John Arne Dahl supervised the team at the University of Oslo, and participated in writing the manuscript. Steve Horvath and Shigemi Matsuyama initiated the study, performed the experiments, analyzed the data, and wrote the manuscript. Steve Horvath performed the DNAm age calculations based on DNAm data. Shigemi Matsuyama proposed and designed the overall strategy of this study and organized the team efforts. Arne Soraas, Mieko Matsuyama, and Shigemi Matsuyama wrote the majority of the manuscript and completed the final version for submission.

## References

[acel12897-bib-0001] Ambatipudi, S. , Horvath, S. , Perrier, F. , Cuenin, C. , Hernandez‐Vargas, H. , Le Calvez‐Kelm, F. , … Herceg, G. (2017). DNA methylome analysis identifies accelerated epigenetic ageing associated with postmenopausal breast cancer susceptibility. European Journal of Cancer, 75, 299–307. 10.1016/j.ejca.2017.01.014 28259012 PMC5512160

[acel12897-bib-0002] Bernitz, J. M. , Daniel, M. , Fstkchyan, Y. S. , & Moore, K. (2017). Granulocyte‐colony stimulating factor mobilizes dormant hematopoietic stem cells without proliferation in mice. Blood, 129, 1901–1912. 10.1182/blood-2016-11-752923 28179275 PMC5383874

[acel12897-bib-0003] Carroll, J. E. , Irwin, M. R. , Levine, M. , Seeman, T. E. , Absher, D. , Assimes, T. , & Horvath, S. (2017). Epigenetic aging and immune senescence in women with insomnia symptoms: Findings from the Women's Health Initiative Study. Biological Psychiatry, 81, 136–144. 10.1016/j.biopsych.2016.07.008 27702440 PMC5536960

[acel12897-bib-0004] Castellano, J. M. , Mosher, K. I. , Abbey, R. J. , McBride, A. A. , James, M. L. , Berdnik, D. , … Wyss‐Coray, T. (2017). Human umbilical cord plasma proteins revitalize hippocampal function in aged mice. Nature, 544, 488–492. 10.1038/nature22067 28424512 PMC5586222

[acel12897-bib-0005] Chen, B. H. , Marioni, R. E. , Colicino, E. , Peters, M. J. , Ward‐Caviness, C. K. , Tsai, P. C. , … Horvath, S. (2016). DNA methylation‐based measures of biological age: Meta‐analysis predicting time to death. Aging (Albany NY), 8, 1844–1865. 10.18632/aging.101020 27690265 PMC5076441

[acel12897-bib-0006] Conboy, M. J. , Conboy, I. M. , & Rando, T. A. (2013). Heterochronic parabiosis: Historical perspective and methodological considerations for studies of aging and longevity. Aging Cell, 12, 525–530. 10.1111/acel.12065 23489470 PMC4072458

[acel12897-bib-0007] Dreger, P. , Suttorp, M. , Haferlach, T. , Loffler, H. , Schmitz, N. , & Schroyens, W. (1993). Allogeneic granulocyte colony‐stimulating factor‐mobilized peripheral blood progenitor cells for treatment of engraftment failure after bone marrow transplantation. Blood, 81, 1404–1407. 7680245

[acel12897-bib-0008] Dugué, P. A. , Bassett, J. K. , Joo, J. E. , Jung, C. H. , Ming Wong, E. , Moreno‐Betancur, M. , … Milne, R. L. (2018). DNA methylation‐based biological aging and cancer risk and survival: Pooled analysis of seven prospective studies. International Journal of Cancer, 142, 1611–1619. 10.1002/ijc.31189 29197076

[acel12897-bib-0009] Eggel, A. , & Wyss‐Coray, T. (2014). A revival of parabiosis in biomedical research. Swiss Medical Weekly, 144, w13914 10.4414/smw.2014.13914 24496774 PMC4082987

[acel12897-bib-0010] Hannum, G. , Guinney, J. , Zhao, L. , Zhang, L. , Hughes, G. , Sadda, S. , … Zhang, K. (2013). Genome‐wide methylation profiles reveal quantitative views of human aging rates. Molecular Cell, 49, 359–367. 10.1016/j.molcel.2012.10.016 23177740 PMC3780611

[acel12897-bib-0011] Hooper, A. T. , Butler, J. M. , Nolan, D. J. , Kranz, A. , Iida, K. , Kobayashi, M. , … Rafii, S. (2009). Engraftment and reconstitution of hematopoiesis is dependent on VEGFR2‐mediated regeneration of sinusoidal endothelial cells. Cell Stem Cell, 4, 263–274. 10.1016/j.stem.2009.01.006 19265665 PMC3228275

[acel12897-bib-0012] Horvath, S. (2013). DNA methylation age of human tissues and cell types. Genome Biology, 14, R115 10.1186/gb-2013-14-10-r115 24138928 PMC4015143

[acel12897-bib-0013] Horvath, S. , & Levine, A. J. (2015). HIV‐1 Infection accelerates age according to the epigenetic clock. The Journal of Infectious Diseases, 212, 1563–1573. 10.1093/infdis/jiv277 25969563 PMC4621253

[acel12897-bib-0014] Horvath, S. , & Ritz, B. R. (2015). Increased epigenetic age and granulocyte counts in the blood of Parkinson's disease patients. Aging (Albany NY), 7, 1130–1142. 10.18632/aging.100859 26655927 PMC4712337

[acel12897-bib-0015] Horvath, S. , Erhart, W. , Brosch, M. , Ammerpohl, O. , von Schonfels, W. , Ahrens, M. , … Hampe, J. (2014). Obesity accelerates epigenetic aging of human liver. Proceedings of the National Academy of Sciences of the United States of America, 111, 15538–15543. 10.1073/pnas.1412759111 25313081 PMC4217403

[acel12897-bib-0016] Horvath, S. , Garagnani, P. , Bacalini, M. G. , Pirazzini, C. , Salvioli, S. , Gentilini, D. , … Franceschi, C. (2015). Accelerated epigenetic aging in Down syndrome. Aging Cell, 14, 491–495. 10.1111/acel.12325 25678027 PMC4406678

[acel12897-bib-0017] Levine, M. E. , Hosgood, H. D. , Chen, B. , Absher, D. , Assimes, T. , & Horvath, S. (2015). DNA methylation age of blood predicts future onset of lung cancer in the women's health initiative. Aging (Albany NY), 7, 690–700. 10.18632/aging.100809 26411804 PMC4600626

[acel12897-bib-0018] Maierhofer, A. , Flunkert, J. , Oshima, J. , Martin, G. M. , Haaf, T. , & Horvath, S. (2017). Accelerated epigenetic aging in Werner syndrome. Aging, 9, 1143–1152. 10.18632/aging.101217 28377537 PMC5425119

[acel12897-bib-0019] Marioni, R. E. , Shah, S. , McRae, A. F. , Chen, B. H. , Colicino, E. , Harris, S. E. , … Deary, I. J. (2015). DNA methylation age of blood predicts all‐cause mortality in later life. Genome Biology, 16, 25 10.1186/s13059-015-0584-6 25633388 PMC4350614

[acel12897-bib-0020] Panch, S. R. , Szymanski, J. , Savani, B. N. , & Stroncek, D. F. (2017). Sources of hematopoietic stem and progenitor cells and methods to optimize yields for clinical cell therapy. Biology of Blood and Marrow Transplantation, 23, 1241–1249. 10.1016/j.bbmt.2017.05.003 28495640

[acel12897-bib-0021] Quach, A. , Levine, M. E. , Tanaka, T. , Lu, A. T. , Chen, B. H. , Ferrucci, L. , … Horvath, S. (2017). Epigenetic clock analysis of diet, exercise, education, and lifestyle factors. Aging (Albany NY), 9, 419–446. 10.18632/aging.101168 28198702 PMC5361673

[acel12897-bib-0022] Russell, N. H. , Hunter, A. , Rogers, S. , Hanley, J. , & Anderson, D. (1993). Peripheral blood stem cells as an alternative to marrow for allogeneic transplantation. The Lancet, 341, 1482–2000. 10.1016/0140-6736(93)90929-B 8099182

[acel12897-bib-0023] Spolnicka, M. , Piekarska, R. Z. , Jaskula, E. , Basak, G. W. , Jacewicz, R. , Pieta, A. , … Płoski, R. (2016). Donor age and C1orf132/MIR29B2C determine age‐related methylation signature of blood after allogeneic hematopoietic stem cell transplantation. Clinical Epigen etics, 8, 93 10.1186/s13148-016-0257-7 PMC5012039 27602173

[acel12897-bib-0024] Stölzel, F. , Brosch, M. , Horvath, S. , Kramer, M. , Thiede, C. , von Bonin, M. , … Bornhäuser, M. (2017). Dynamics of epigenetic age following hematopoietic stem cell transplantation. Haematologica, 102, e321–e323. 10.3324/haematol.2016.160481 28550187 PMC5541887

[acel12897-bib-0025] Thiede, C. , Florek, M. , Bornhauser, M. , Ritter, M. , Mohr, B. , Brendel, C. , … Neubauer, A. (1999). Rapid quantification of mixed chimerism using multiplex amplification of short tandem repeat markers and fluorescence detection. Bone Marrow Transplantation, 23, 1055–1060. 10.1038/sj.bmt.1701779 10373073

[acel12897-bib-0026] Thomas, E. D. , Lochte, H. L. Jr. , Lu, W. C. , & Ferrebee, J. W. (1957). Intravenous infusion of bone marrow in patients receiving radiation and chemotherapy. The New England Journal of Medicine, 257, 491–496. 10.1056/NEJM195709122571102 13464965

[acel12897-bib-0027] Thomas, E. D. , Buckner, C. D. , Clift, R. A. , Fefer, A. , Johnson, F. L. , Neiman, P. E. , … Weiden, P. L. (1979). Marrow transplantation for acute nonlymphoblastic leukemia in first remission. The New England Journal of Medicine, 301, 597–599. 10.1056/NEJM197909133011109 381925

[acel12897-bib-0028] Weidner, C. I. , & Wagner, W. (2014). The epigenetic tracks of aging. Biological Chemistry, 395, 1307–1314. 10.1515/hsz-2014-0180 25205717

[acel12897-bib-0029] Weidner, C. I. , Ziegler, P. , Hahn, M. , Brummendorf, T. H. , Ho, A. D. , Dreger, P. , & Wagner, W. (2015). Epigenetic aging upon allogeneic transplantation: The hematopoietic niche does not affect age‐associated DNA methylation. Leukemia, 29, 985–988. 10.1038/leu.2014.323 25388956

