# Supplementary figures and images for: Epigenetic age is a cell‐intrinsic property in transplanted human hematopoietic cells
Source: Aging Cell. 2019 Feb 2;18(2):e12897. doi: 10.1111/acel.12897 (PMC6413751; doi:10.1111/acel.12897)

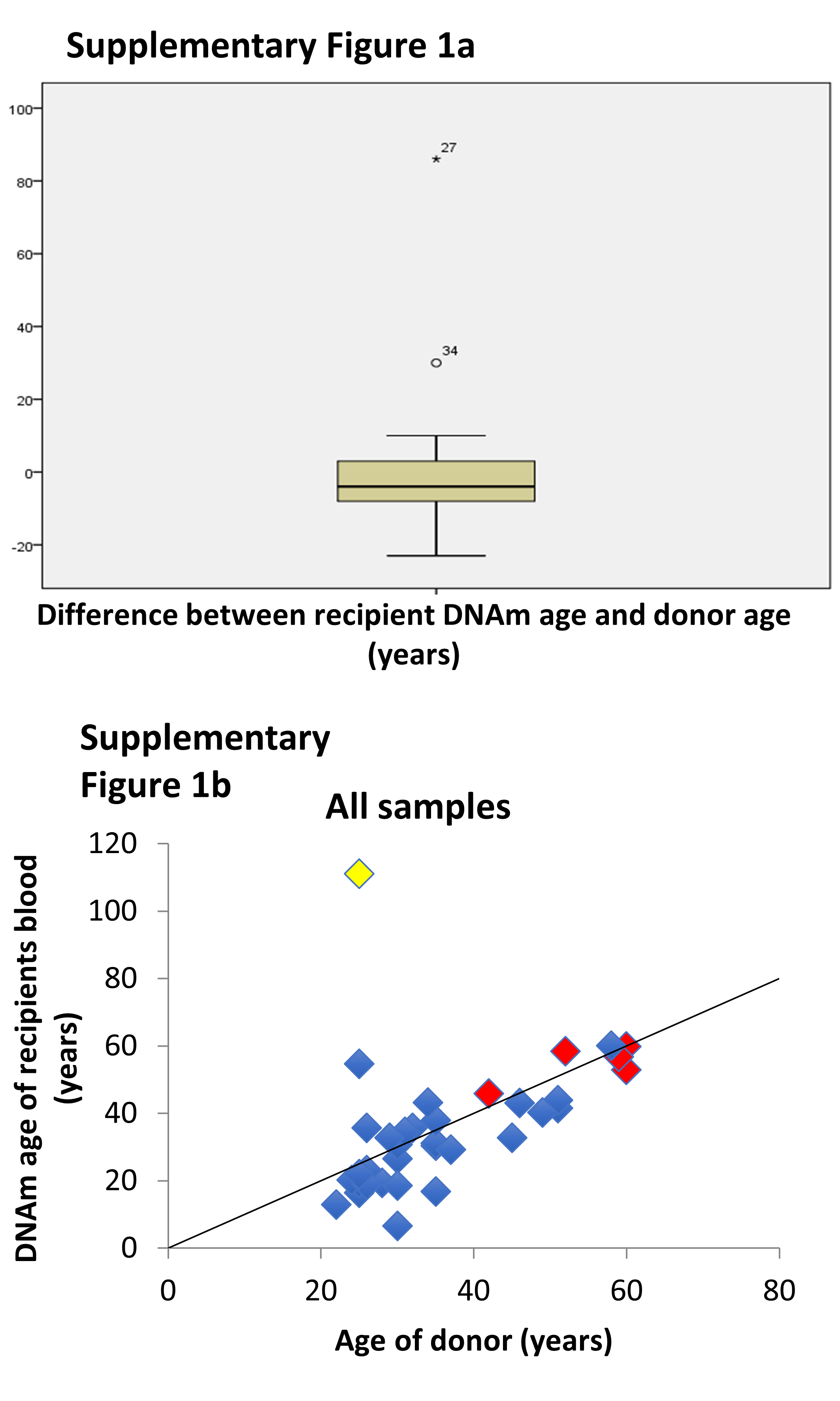

Supplement: Supplementary file 1 [file ACEL-18-e12897-s001.PNG]
